# Policy Implications of the Active Aging Framework in Poland

**DOI:** 10.1093/geroni/igab046.1663

**Published:** 2021-12-17

**Authors:** Jolanta Perek-Białas, Maria Varlamova

**Affiliations:** Jagiellonian University, Krakow, Malopolskie, Poland

## Abstract

Poland's relatively young population in the past, is aging rapidly, which provokes a growing interest in the realisation of the older population's potential within the framework of the concept of active ageing. From 2012, when the first Governmental Program on Social Participation (ASOS 2012-2013) was introduced, the active aging framework remains one of the dominant strategies in developing and implementing social policies for the older generation. In the current paper, the focus is made on the employment and social participation of older citizens policies, showing the considerable gap in the prioritisation and hence in the outcomes, highlighted both by Active Ageing Index (AAI) indicators and more in-depth analysis. We will discuss the perceived risks and pitfalls of the current long-term ageing policy approach in Poland and provide recommendations for improvement.

